# Properties of SF-6D when longitudinal data from 16,398 spine surgery procedures is applied to 9 national SF-6D value sets

**DOI:** 10.1080/17453674.2021.1915524

**Published:** 2021-04-23

**Authors:** Anders Joelson, Freyr Gauti Sigmundsson, Jan Karlsson

**Affiliations:** a Department of Orthopedics, Orebro University School of Medical Sciences and Orebro University Hospital, Orebro;; b University Health Care Research Center, Faculty of Medicine and Health, Orebro University, Orebro, Sweden

## Abstract

Background and purpose — There are several national value sets for SF-6D. For studies conducted in countries without a country-specific value set the authors may use a value set from a neighboring or culturally similar county. We evaluated the consequences of using different national value sets in SF-6D index-based outcome analyses.

Patients and methods — Patients surgically treated for lumbar spinal stenosis or lumbar disk herniation between 2007 and 2017 were recruited from the national Swedish spine register. 16,398 procedures were eligible for analysis. The SF-6D health states were coded to SF-6D preference indices using value sets for 9 countries. The SF-6D index distributions were then estimated with kernel density estimation. The change in SF-6D index before and after treatment was evaluated with the standardized response mean (SRM).

Results — There was a marked variability in mean and shape for the resulting SF-6D index distributions. There were considerable differences in SF-6D index distribution shape before and after treatment using the same value set. The effect sizes of 2-year change (SRM) were in most cases similar when the 9 value sets were applied on pre- and post-treatment data.

Interpretation — We found a marked variability in SF-6D index distributions when a single large data set was applied to 9 national SF-6D value sets. Consequently, we recommend that SF-6D index data from studies conducted in countries without country-specific SF-6D value sets is interpreted with caution.

The Short Form 6-dimensional instrument (SF-6D) (Brazier et al. 1998, 2002, Brazier and Roberts 2004) and the EuroQol 5-dimensional instrument (EQ-5D) (EuroQol Group 1990) are 2 similar multilevel preference-based measures for assessment of general health. The instruments are primarily used for calculation of quality adjusted life years (QALYs) in economic evaluation of health interventions. The two instruments use different national value sets (also called tariffs) to adjust for national differences in experience of health-related quality of life (HRQoL). For EQ-5D, previous studies have raised the concern that data derived from different national value sets is not fully comparable. Van Dongen et al. (2021) estimated EQ-5D index values for 16 country-specific value sets and found that the use of different country-specific value sets has an impact on cost–utility outcomes. This finding is of particular importance when conducting studies in countries without country- or region-specific value sets, as the results may depend on the choice of value set. For SF-6D, data on national variations in SF-6D index distribution is lacking.

We evaluated the consequences of using different national value sets in SF-6D index-based outcome analyses. We applied a single large longitudinal SF-6D data set to several national SF-6D value sets to explore differences in SF-6D index distributions and effect sizes before and after treatment. We used SF-36 data (collected before and 2 years after surgery) from the national Swedish spine register (Swespine) for 2 of the most common spinal surgery diagnoses: spinal stenosis and disk herniation.

## Patients and methods

### Study design

This study was a register study with prospectively collected longitudinal data from the national Swedish spine register, Swespine. Swespine was launched in 1992, the national coverage is 90% of the spine units in Sweden, and the follow-up rate is 75–80% (Strömqvist et al. 2013).

### SF-6D

SF-6D is a multilevel preference-based measure for assessment of general health (Brazier et al. 1998, 2002, 2020 Brazier and Roberts 2004,). The 6 dimensions are: physical functioning (PF), role limitations (RL), social functioning (SF), pain (P), mental health (MH), and vitality (VT). SF-6D is based on 11 items of the Medical Outcomes Study 36-item short-form health survey (SF-36) (Ware and Sherbourne 1992). PF and P have 6 response options, SF, MH, and VT have 5 response options, and RL has 4 response options. The response options are coded on an ordinal scale from 1 to 4–6 (1 being the best). The answers are assembled to a 6-digit health state reflecting the score on each dimension (in total 6 × 4×5 × 6×5 × 5 = 18,000 states, 111111 being the best and 645655 the worst). There are 2 major versions of SF-6D, the SF-36 version (Brazier et al. 2002) (used in this study) and the SF-12 version (Brazier and Roberts 2004).

Each health state can be coded to a preference-based index (hereafter denoted as SF-6D index) using a value set (tariff). The SF-6D index usually ranges between 0 (states equal to death) and 1 (full health) but some SF-6D indices also include values less than 0 (health states worse than death). There are several national value sets for coding health states to SF-6D indices. For our study, we wanted broad coverage of several continents and we selected the following 9 national value sets for our investigation: Australia (Norman et al. 2014), Brazil (Cruz et al. 2011), China (Lam et al. 2008), Spain (Abellan Perpinan et al. 2012), Hong Kong (McGhee et al. 2011), Lebanon (Kharroubi et al. 2020), Portugal (Ferreira et al. 2010), the UK (Brazier et al. 2002), and the USA (Craig et al. 2013). Our literature review also identified a Dutch value set (Jonker et al. 2018) and a Japanese value set (Brazier et al. 2009). The Dutch value set was excluded from our analysis since it was based on SF-12. The Japanese value set was excluded because of inferior performance compared with the UK value set in terms of inconsistencies (worsening in dimension did not lower the index) and prediction errors (capability to predict the index of a health state).

### Patient data set

Patients were recruited from the national Swedish spine register (Swespine). 52,560 procedures for surgical treatment of lumbar spinal stenosis and lumbar disk herniation between 2007 and 2017 are included in the register. Preoperative or 2-year postoperative SF-6D data was incomplete for 36,162 procedures, which gave 16,398 (31%) procedures eligible for analysis (Table 1).

**Table 1.** Characteristics of the study population

	Spinal stenosis	Disk herniation
Factor	n = 8,888	n = 7,510
Age, mean (SE)	66.6 (0.11)	45.7 (0.15)
BMI (SE)	27.8 (0.05)	26.4 (0.05)
Females, n (%)	3,999 (45)	3,345 (45)
Smokers, n (%)	826 (9)	950 (13)

### Data transformation

SF-36 data was collected from Swespine (preoperative and 2-year postoperative data). SF-36 data was converted to SF-6D and then coded to SF-6D indices using the 9 national value sets. The conversion from health state to index was implemented in the language R (R Foundation for Statistical Computing, Vienna, Austria) using the models given in the references for the 9 value sets (see Supplementary Appendix for notes on implementation).

### General properties

To illustrate general properties of the 9 national value sets, we computer generated a data set consisting of all 18,000 SF-6D health states (111111 to 645655) and then estimated the SF-6D index distribution for all 9 national value sets with kernel density estimation.

### Statistics

The effect size (the difference in means in terms of standard deviations) was evaluated using the standardized response mean (SRM) for paired data (the difference in means divided by the standard deviation of the difference) (Fayers and Machin 2016, Table 20.1, p. 535). An approximate 95% confidence interval (CI) for SRM is given by Fayers and Machin (2016, eq. 20.4, p. 542 and Table 20.1, p. 535). The SRM was interpreted as follows (Fayers and Machin 2016, p. 499): < 0.2 no effect, 0.2–0.4 small effect, 0.5–0.7 moderate effect, > 0.7 large effect.

The distribution of a random variable specifies how the values of the random variable are distributed for all possible values of the random variable. We used kernel density estimation with Gaussian kernels to estimate the distribution of the SF-6D index (hereafter denoted as the SF-6D index distribution). See Supplementary Appendix for details on kernel density estimation.

### Ethics, data sharing, funding, and potential conflicts of interest

The study was approved by the regional ethical review board (registration number: 2020-03557). Data are available from the national Swedish spine register (Swespine) after approval by a Swedish regional ethical review board and approval by the Swespine board. There was no external source of funding for this study. The authors declare no conflicts of interest.

## Results

**Figure 1.**
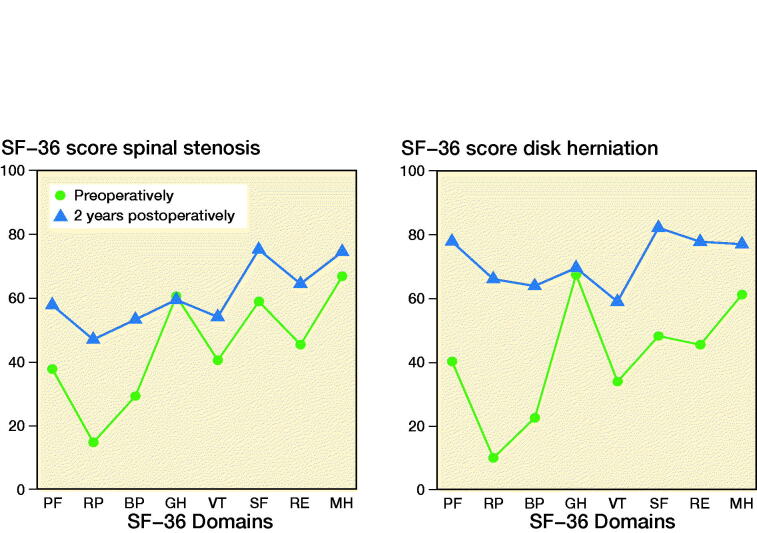
SF-36 scores for spinal stenosis (n = 8,888) and disk herniation (n = 7,510) preoperatively (green dots) and 2 years postoperatively (blue triangles). The standard errors were less than 0.6 for all domains. PF = physical functioning, RP = role limitation due to physical problems, BP = bodily pain, GH = general health, VT = vitality, SF = social functioning, RE = role limitations due to emotional problems, MH = mental health.

SF-36 data preoperatively and 2 years postoperatively are presented in Figure 1. Outcome was improved for all domains except for general health (GH).

**Figure 2.**
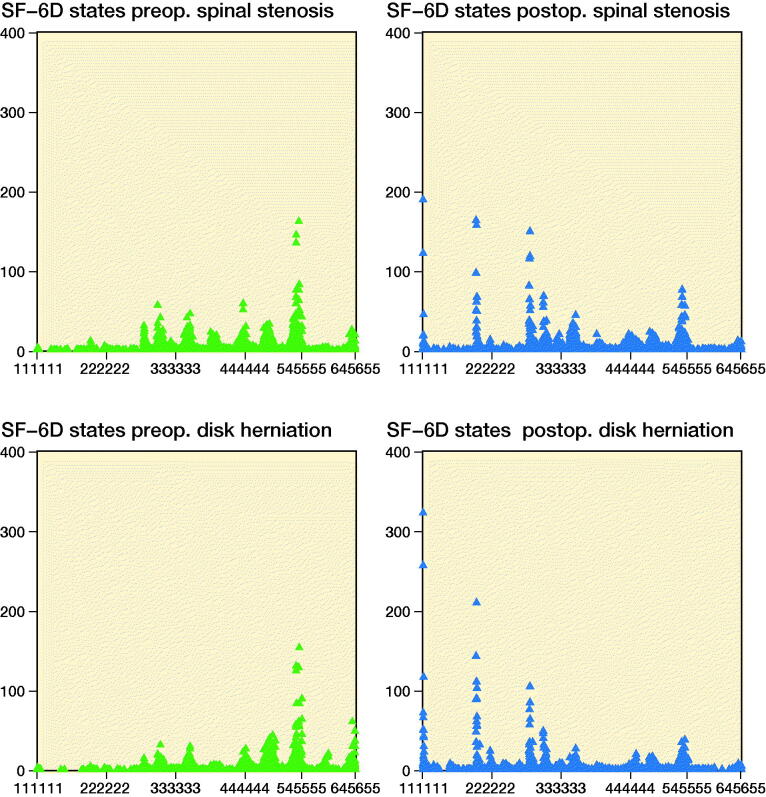
Frequency of SF-6D states (triangles) before and after surgical treatment for spinal stenosis (n = 8,888) and disk herniation (n = 7,510).

The SF-6D state distributions preoperatively and postoperatively are presented in Figure 2. The distribution of health states showed some clustering both preoperatively and postoperatively. The health states shifted towards lower values postoperatively for both spinal stenosis and disk herniation. The best possible health state (111111) was the most common state 2 years postoperatively for both spinal stenosis (n = 189, 2.1%) and disk herniation (n = 323, 4.3%). The worst possible health state (645655) was uncommon (< 0.5% of the health states).

**Figure 3.**
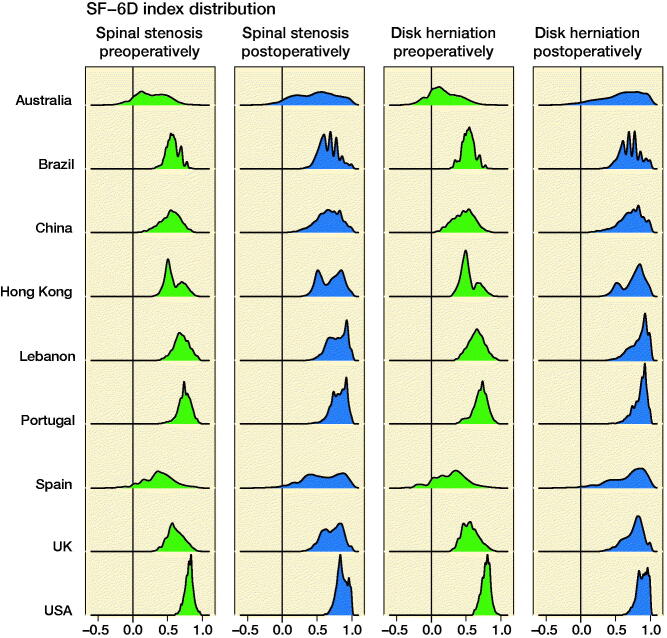
Kernel density estimates of the SF-6D index distributions for 9 different national SF-6D value sets based on data from patients treated for spinal stenosis (n = 8,888) and patients treated for disk herniation (n = 7,510).

The estimation of the SF-6D index distributions for the 9 national value sets is given in Figure 3. There were marked differences in distribution shapes (unimodal, bimodal, and multimodal).

**Figure 4.**
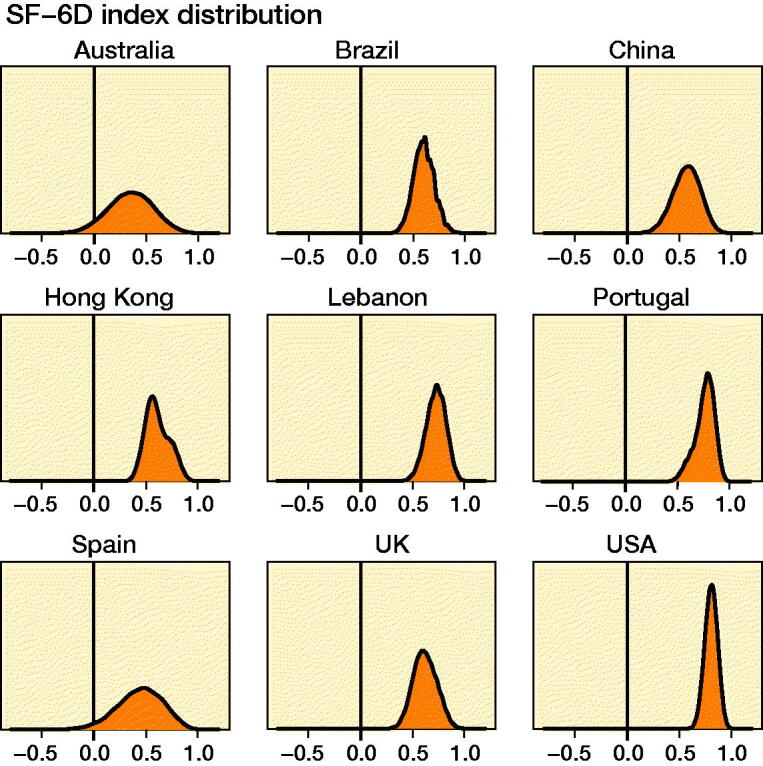
Kernel density estimates of the SF-6D index distributions for 9 different national SF-6D value sets based on a computer-generated data set consisting of all possible 18,000 SF-6D health states (111111 to 645655).

The estimation of the SF-6D index distributions for all 18,000 SF-6D health states (111111 to 645655) is shown in Figure 4. There were substantial differences primarily in widths but also in shapes of the distributions.

Table 2 summarizes the mean and median SF-6D indices for the different national SF-6D value sets. The mean and median values were similar for a given national value set. There were, however, substantial differences between the national value sets. The effect sizes of 2-year change (SRM) were in most cases similar when the 9 value sets were applied to pre- and post-treatment data. For spinal stenosis the patients had moderate treatment effects and for disk herniation the treatment effects were large.

**Table 2.** Preop and postop SF-6D indices for 16,398 spine surgery patients based on different national value sets

Country	Preop		Postop		Effect size SRM (95% CI)
	Mean (SE)	Median (IQR)	Mean (SE)	Median (IQR)	
Spinal stenosis (n = 8,888)					
Australia	0.27 (0.0024)	0.26 (0.10–0.44)	0.48 (0.0031)	0.50 (0.24–0.70)	0.76 (0.74–0.78)
Brazil	0.58 (0.0010)	0.57 (0.51–0.63)	0.67 (0.0014)	0.67 (0.57–0.78)	0.70 (0.68–0.73)
China	0.54 (0.0015)	0.55 (0.45–0.64)	0.67 (0.0019)	0.68 (0.55–0.81)	0.72 (0.70–0.75)
Spain	0.37 (0.0023)	0.38 (0.24–0.51)	0.57 (0.0029)	0.58 (0.38–0.81)	0.75 (0.73–0.78)
Hong Kong	0.59 (0.0013)	0.55 (0.50–0.69)	0.70 (0.0017)	0.72 (0.54–0.84)	0.71 (0.69–0.73)
Lebanon	0.70 (0.0012)	0.70 (0.63–0.78)	0.79 (0.0014)	0.81 (0.69–0.92)	0.71 (0.69–0.74)
Portugal	0.75 (0.0010)	0.74 (0.69–0.81)	0.82 (0.0012)	0.83 (0.74–0.92)	0.67 (0.65–0.69)
UK	0.61 (0.0013)	0.61 (0.54–0.70)	0.72 (0.0015)	0.73 (0.61–0.84)	0.73 (0.71–0.76)
USA	0.81 (0.0006)	0.81 (0.77–0.85)	0.86 (0.0008)	0.86 (0.81–0.93)	0.69 (0.66–0.71)
Disk herniation (n = 7,510)					
Australia	0.18 (0.0025)	0.15 (0.02–0.33)	0.59 (0.0034)	0.64 (0.40–0.83)	1.33 (1.29–1.36)
Brazil	0.54 (0.0011)	0.54 (0.48–0.59)	0.71 (0.0016)	0.69 (0.61–0.78)	1.18 (1.15–1.21)
China	0.47 (0.0018)	0.47 (0.36–0.58)	0.73 (0.0020)	0.75 (0.63–0.84)	1.29 (1.26–1.32)
Spain	0.27 (0.0026)	0.30 (0.12–0.43)	0.67 (0.0030)	0.74 (0.51–0.87)	1.34 (1.31–1.38)
Hong Kong	0.54 (0.0014)	0.50 (0.46–0.62)	0.76 (0.0018)	0.81 (0.66–0.87)	1.30 (1.27–1.33)
Lebanon	0.65 (0.0013)	0.65 (0.57–0.73)	0.84 (0.0015)	0.88 (0.76–0.94)	1.30 (1.27–1.33)
Portugal	0.71 (0.0012)	0.72 (0.65–0.78)	0.86 (0.0012)	0.88 (0.81–0.93)	1.21 (1.18–1.24)
UK	0.56 (0.0014)	0.56 (0.47–0.64)	0.77 (0.0016)	0.80 (0.68–0.86)	1.29 (1.26–1.32)
USA	0.79 (0.0007)	0.79 (0.74–0.83)	0.89 (0.0009)	0.90 (0.84–0.96)	1.17 (1.14–1.20)

## Discussion

The primary purpose of this study was to investigate whether the choice of national value set had any impact on the SF-6D indices. To our knowledge our study is the first comparison of several different SF-6D national value sets based on a large longitudinal data set.

We found a marked variability in SF-6D index distributions when a single large data set was applied to 9 national SF-6D value sets (Figure 3). There were differences between the different value sets and also differences between distributions before and after surgery using the same value set. This means that it is not only the mean/median SF-6D index that may change after a medical intervention: the entire shape of the distribution may be different after an intervention. This finding has consequences for the statistical inference on paired data when SF-6D index before and after a medical intervention is evaluated (assumptions on normality and/or variance equality are violated).

There were marked differences in the SF-6D indices between the different value sets, both before and after surgery (Table 2). The effect sizes of 2-year change (SRM), however, were in most cases similar when the 9 value sets were applied to pre- and post-treatment data (see Table 2). This means that evaluations of treatment effects, in some cases, seem to be less sensitive to differences in value sets than the absolute index values. The SRM is often used to evaluate responsiveness to changes in psychometric evaluations of HRQoL instruments. The SRMs of our study are similar to the SRM reported by Carreon et al. (2013) for a cohort of 1,104 patients who underwent lumbar decompression and fusion. Angst et al. (2017) suggested that the SRM can be used as an approximate estimate of the minimal clinically important difference (MCID) (cut off SRM 0.3–0.5) if an MCID evaluation study is not available for reasons of cost, time, or other constraints. For our data, the minimum SRM was 0.67 (Portuguese value set), which suggests that the improvements in SF-6D, irrespective of choice of value set, are clinically significant for spinal stenosis surgery and disk herniation surgery.

QALY gain calculation is different from effect size calculation because QALY gain, as opposed to effect size, is often calculated in terms of differences in means and not in terms of differences in standard deviations (Sassi 2006). For example, in our data for spinal stenosis (see Table 2), the UK mean SF-6D index increases 0.11 points (from 0.61 to 0.72) while the corresponding US index increment is 0.05 points. Consequently, using different SF-6D value sets on exactly the same data set might result in substantial differences in QALY gain. This finding is of particular importance when conducting studies in countries without country- or region-specific value sets, as the results may depend on the choice of value set.

Few previous studies have compared different national SF-6D value sets. The validation of the US SF-6D value set (Craig et al. 2013) showed good correlation when applying data of 8,428 respondents to the US and the UK value sets. In contrast, our distribution estimates showed marked differences in shape and widths between the US and UK distributions. A validation of the Lebanese SF-6D value set (Kharroubi et al. 2020) found marked differences between the Lebanese SF-6D model and the UK SF-6D model. Also, the predictive ability of the Lebanese model was superior to the UK model when applying Lebanese data. The differences in models are confirmed by our distribution estimates (see Figure 3). Ferraz et al. (2019) applied the Brazilian and the UK preference weights on a Brazilian urban population and found only small quantitative differences. In contrast, our study found marked differences in distributions (see Figure 3) and effect sizes (see Table 2) when comparing the Brazilian and UK value sets. One possible explanation for the marked differences in our study is that patients from different countries may fill out the SF-6D differently while being in the same health condition. Consequently, there may be an imbalance between the response pattern of Swedish patients and, e.g., the UK value set. This imbalance might partly explain the differences in SF-6D index distributions found in our study.

The estimated SF-6D index distributions based on the computer-generated data set consisting of all 18,000 SF-6D health states (Figure 4) explain some of the properties of the distributions given in Figure 3, e.g., the large width of the Australian distribution and the limited width of the US distribution. Some properties, however, are more difficult to understand, e.g., the bimodality of the Hong Kong distribution. The clustering of SF-6D states illustrated in Figure 2 seems to have only a minor impact on the SF-6D index distribution.

Our findings should be evaluated in the light of several limitations. First, we recognize the inherent limitations of register data, e.g., lack of confounder information, missing data, or unknown data quality (Thygesen and Ersbøll 2014). Second, the data were limited to spine surgery patients, i.e., persons with problems mainly related to the musculoskeletal system. Third, the conversion of the 18,000 SF-6D health states to the SF-6D index represents a nonlinear multivariate transformation on discrete, sometime clustered, data. The analysis of such a model is mathematically challenging. In favor of more complex mathematical methods, we used descriptive statistics and graphical representations to explore our data. Fourth, we implemented SF-6D using the specification given in the paper by Brazier et al. (2002). The specification has inconsistencies that may introduce systematic errors in our SF-6D data (cf. Supplementary Appendix). Fifth, data were complete for 31% of the procedures.

In conclusion, we found a marked variability in SF-6D index distributions when a single large data set was applied to 9 national SF-6D value sets. Consequently, studies that aggregate international data, e.g., meta-analyses, may produce misleading results if the underlying differences in SF-6D index distributions are inadequately handled. On the basis of the results of our study we recommend that SF-6D index data from studies conducted in countries without country or region-specific SF-6D value sets is interpreted with caution.

## Supplementary Material

Supplemental Material

## Supplementary data

The Appendix is available as supplementary data in the online version of this article, http://dx.doi.org/10.1080/17453674. 2021.1915524
