# Supplementary material for: Properties of SF-6D when longitudinal data from 16,398 spine surgery procedures is applied to 9 national SF-6D value sets
Source: Acta Orthop. 2021 Apr 23;92(5):532–7. doi: 10.1080/17453674.2021.1915524 (PMC8519553; doi:10.1080/17453674.2021.1915524)
Supplement: Supplemental Material [file IORT_A_1915524_SM8163.pdf]

Supplementary data

Implementation of SF-6D

The current study used the original version of SF-36 (Ware et al. 1992) and we implemented SF-6D using the specification given in the paper by Brazier et al. (2002). Our paper uses the SF-36 item numbering of Ware et al. (1992). There are some known inconsistencies that have been addressed in the second version of SF-6D (Brazier et al. 2020). We handled these inconsistencies as follows:

SF-6D Physical functioning (SF-36 items 3a, 3b, 3j): Unclear how to handle the case when the patient answers "Limited a lot" on item 3a and "Not limited at all" on items 3b and 3j. We coded this case as SF-6D Physical functioning level 3. Moreover, the order between item 3b and item 3j is unclear. We coded the combination "Limited a lot" on item 3b and "Limited a little" on item 3j as SF-6D level 5.

SF-6D Role limitations (SF-36 items 4c, 5b): No inconsistencies. It would have been clearer if the answers on item 4c and 5b were explicitly stated on all levels of the SF-6D Role limitations domain.

SF-6D Social functioning (SF-36 item 10): No inconsistencies. The scale, however, is reversed on SF-6D compared with SF-36.

SF-6D Pain (SF-36 items 7, 8): Unclear how to handle the case when the patients answer "None" on item 7 and "Very mild" to "Very severe" on item 8. We used the answers on item 8 to code the level of SF-6D Pain in this case.

SF-6D Mental health (SF-36 items 9b, 9f): 6 levels on item 9b and 9f but only 5 levels on SF-6D. We coded "A good bit of the time" as SF-6D Mental health level 3.

SF-6D Vitality (SF-36 item 9e): 6 levels on item 9e but only 5 levels on SF-6D. We coded "A good bit of the time" as SF-6D Vitality level 3.

Kernel density estimation

Histograms are commonly used for estimation of density functions where the height of each bar of the histogram represents the number of data points within the width of the bar. An obvious drawback of histograms is that histograms are inherently discontinuous in shape. Kernel density estimation (KDE) generalizes the idea of histograms and generates smooth continuous density estimates. In short, KDE smooths each data point by means of a smoothing function (called kernel function) and adds them all up to obtain the final density estimate. This means that all data points contribute to the final estimate. A commonly used kernel function is the normal (Gaussian) function $f(x) = (\mathrm{sqrt}(2\varpi))^{-1}\exp(-x^2/2)$. An illustrative example of KDE with Gaussian kernels is given in Figure 12.6 p. 354 in Rizzo (2019).
